# Mechanosensitivity of the BK Channels in Human Glioblastoma Cells: Kinetics and Dynamical Complexity

**DOI:** 10.1007/s00232-018-0044-9

**Published:** 2018-08-09

**Authors:** Agata Wawrzkiewicz-Jałowiecka, Paulina Trybek, Łukasz Machura, Beata Dworakowska, Zbigniew J. Grzywna

**Affiliations:** 1 Department of Physical Chemistry and Technology of Polymers, Faculty of Chemistry, Silesian University of Technology, Gliwice, Poland; 2 Division of Computational Physics and Electronics, Institute of Physics, Silesian Centre for Education and Interdisciplinary Research, University of Silesia in Katowice, Katowice, Poland; 3 Division of Biophysics, Department of Physics, Warsaw University of Life Sciences – SGGW, Warszawa, Poland

**Keywords:** BK channels, Mechanosensitivity, Glioblastoma cells

## Abstract

BK channels are potassium selective and exhibit large single-channel conductance. They play an important physiological role in glioma cells: they are involved in cell growth and extensive migrating behavior. Due to the fact that these processes are accompanied by changes in membrane stress, here, we examine mechanosensitive properties of BK channels from human glioblastoma cells (gBK channels). Experiments were performed by the use of patch-clamp method on excised patches under membrane suction (0–40 mmHg) at membrane hyper- and depolarization. We have also checked whether channel’s activity is affected by possible changes of membrane morphology after a series of long impulses of suction. Unconventionally, we also analyzed internal structure of the experimental signal to make inferences about conformational dynamics of the channel in stressed membranes. We examined the fractal long-range memory effect (by R/S Hurst analysis), the rate of changes in information by sample entropy, or correlation dimension, and characterize its complexity over a range of scales by the use of Multiscale Entropy method. The obtained results indicate that gBK channels are mechanosensitive at membrane depolarization and hyperpolarization. Prolonged suction of membrane also influences open–closed fluctuations—it decreases channel’s activity at membrane hyperpolarization and, in contrary, increases channel’s activity at high voltages. Both membrane strain and its “fatigue” reduce dynamical complexity of channel gating, which suggest decrease in the number of available open conformations of channel protein in stressed membranes.

## Introduction

BK channels (MaxiK) are highly selective for potassium ions and distinguish itself from other channel types by large single-channel conductance (~ 100–300 pS) (Cox 2007; Cui et al. 2008). They are activated mainly by membrane depolarization and elevated cytosolic concentration of calcium ions; however, there are also other factors that can affect its gating: intracellular Mg^2+^, protons, heme, ethanol, carbon monoxide, PIP_2_, Omega-3, and temperature (Wawrzkiewicz-Jałowiecka et al. 2017; Yang et al. 2015). It turns out that in many cell types, e.g., neuroepithelial, skeletal muscle, smooth muscle, trabecular meshwork cells, BK channels exhibit also strong mechanosensitivity (Allard et al. 2000; Davidson 1993; Gasull et al. 2003; Kirber et al. 1992; Mienville et al. 1996; Mobasheri et al. 2010; Tang et al. 2003; Zhao et al. 2010).

In current studies, we examine whether mechanical strain of cell membrane affects BK channel gating in human glioblastoma cells. Glioblastoma is the most aggressive primary brain tumor (Louis et al. 2007). Cell growth and extensive migrating behavior of glioblastoma cells are facilitated by the activity of BK channels (Weaver et al. 2006). Moreover, BK channels are overexpressed in malignant gliomas (in comparison with nonmalignant cortical tissues), and the expression level correlates positively with the malignancy grade of the tumor (Liu et al. 2002; Ransom and Sontheimer 2001). MaxiK channels are expressed in specific isoforms in glioma cells which have slightly different characteristics than in other locations [for example, they are more sensitive to cytosolic intracellular [Ca^2+^] (Abdullaev et al. 2010; Ransom et al. 2002)], so they are sometimes called gBK channels.

Glioma cells undergo shape and volume changes during their invasive migration in crowded environment. We are convinced that gBK channels can be involved in that process according to three separate mechanisms:

1. Due to the fact that ions exert an effective osmotic pressure, the distribution of ions affects water flow across cell membrane and thus cell volume (Baumgarten and Feher 2001). Thus, in terms of overexpression of gBK channels in gliomas, these channels can detrimentally affect osmosis and regulation of cell volume.
2. Mechanosensitive channels are involved in mechanostransduction during cell shape and volume changes. Thus, the postulated mechanosensitivity of gBK channels could enhance their physiological meaning in gliomas.
3. Ion channels are structurally anchored in the membrane by specific components of the cytoskeleton (Luna and Hitt 1992) and their interaction may help to regulate cell volume (Baumgarten and Feher 2001). Deformations or reorganization of the cytoskeleton during alterations in cell volume or shape may affect functioning of gBK channels, which, in consequence, should affect the effectiveness of cell migration.

Because overexpression of BK channels in glioma cells is well described in literature, in the current work, we focus on the two last points. Namely, we investigate mechanosensitive properties of gBK channels. In that aim, single-channel patch-clamp recordings are analyzed in terms of mechanical stress of the membrane (induced by membrane suction) at membrane depolarization and hyperpolarization. We also extended the analysis of BK channel mechanosensitivity beyond the level of impact of mechanical strain within cell membrane on gBK channel gating. Namely, we also examine the role of changes in cytoskeleton structure (its bonds with cell membrane) in channel activity. In that aim, analysis of channel’s activity was carried out for single-channel currents measured on excised patches at relaxed membrane between relatively long impulses of membrane suction. Excised patches reduce the impact of cytoskeleton which in cell-attached configuration could prevent excessive buckling of the membrane during suction (Suchyna et al. 2009). Even if some remains of the cytoskeleton are anchored to the patch of membrane, they can be broken down with repeated suction pulses (Sachs 2010; Suchyna et al. 2009). This affects membrane morphology rendering it more “loose” and liable to deformations (Sachs 2010). Here, we perform series of experiments in an alternate pressure mode, where measurements of single-channel current on one patch under no difference in pressure on either side of membrane are intertwined with relatively long pulses of membrane suction (over 1 min).

Not only kinetic properties of gating were studied but we also aim to get some inferences about conformational dynamics of gBK channels at applied experimental conditions. Our research may allow to infer about changes in the number of channel states (available from geometrical and energetic point of view) at different experimental conditions (membrane suction or weakened interactions with cytoskeleton).

In that aim, we analyzed internal structure of the signal—long-range correlations and its entropy. As suggested in the literature, an interesting feature of the series of adjacent dwell time of BK channel states is the long-term memory measured by Hurst exponent using R/S analysis (Barbosa et al. 2007; Campos de Oliveira et al. 2006; Varanda et al. 2000; Wawrzkiewicz et al. 2012; Wawrzkiewicz-Jałowiecka et al. 2017). This characteristics seem to be an inherent property of the system implied by conformational dynamics of the channel gate, which is not fractured by external conditions like membrane potential, Ca^2+^ concentration, or temperature (Barbosa et al. 2007; Wawrzkiewicz-Jałowiecka et al. 2017). Here, we investigate whether mechanical membrane strain may exert a considerable effect on long-range correlations due to possible small changes in spatial arrangement channel domains which, in turn, may influence energetic landscape of conformational space and, in consequence, gating.

For the comprehensive evaluation of the sequences of the dwell times of variability in different experimental conditions, also other measures of dynamical complexity were implemented. Traditional analysis, mainly based on the conventional statistics of signals components brings only limited knowledge on the actual process hidden behind the analyzed data. The parameters that characterize the complexity of the system are closely related to its ability to adapt in the ever-changing environment. The decrease of the level of such complexity may be caused by occurrences of pathological states that significantly stand out from the normal system’s conditions. The information-based measures such as Sample Entropy (*SE*) and Multiscale Entropy (*MSE*) give a knowledge about the level of uncertainty in the context of the signal dynamics and has been successfully applied in the biological data analysis (Gao et al. 2015; Marken et al. 2016; Richman and Moorman 2000). Another feature that gets a broad insight into complex dynamics of the acquired data is the Correlation Dimension (*D*_corr_), which is well established in chaos theory (Ding et al. 1993). This measure is also extensively used in the biological data interpretation (Iannaccone and Khokha 1996). Nevertheless, it is worth to emphasize that this approach may not be conventional and most of the previous studies of dwell-time sequences do not take into account the results of analysis by nonlinear methods.

## Methods

### Cell Culture

Human glioblastoma cells (U-87 MG cell line) were cultured on Petri dishes in Dulbecco’s modified Eagle’s medium (HyClone) supplemented with 2 mM l-glutamine (Gibco), 10% fetal bovine serum (Gibco), 100 units/ml penicillin, and 100 µg/ml streptomycin (Sigma). The cultures were incubated at 37 °C in 5% CO_2_-enriched air.

### Electrophysiological Recordings

Standard patch-clamp techniques were used to record single-channel currents from excised membrane patches in inside-out configuration. In all experiments, the same solution was used in bath and pipette which contained the following: 2 mM CaCl_2_, 2 mM EGTA, 10 mM HEPES, 135 mM potassium gluconate, and pH was adjusted to 7.3. Ion currents were recorded using an Axopatch 200B amplifier (Axon Instruments), low-pass filtered at 10 kHz and transferred to a computer at a sampling frequency of 20 kHz using Clampex 7 software (Axon Instruments). The error of channel current was Δ*I* = 5 × 10^−4^ (pA) and the time resolution was Δ*t* = 1 × 10^−4^ (s).

To analyze the impact of membrane tension in terms of membrane hyperpolarization and depolarization, analogous experiments were performed at fixed pipette potentials *U*_m_ = − 50 (mV) and *U*_m_ = + 50 (mV).

Mechanosensitivity of BK channels was examined by application of suction to patch pipette in the range of 0–40 mmHg with Δ*p* = 10 mmHg step. Experiments were performed in two different modes:

- with gradual monotonic change of membrane suction (*p*_1_ = 0 (mmHg), *p*_2_ = 10 (mmHg), *p*_3_ = 20 (mmHg) etc.); and
- in alternate suction mode—measurements under membrane suction are separated by recordings where membrane is in a resting state (*p*_1_ = 0 (mmHg), *p*_2_ = 10 (mmHg), *p*_3_ = 0 (mmHg), *p*_4_ = 20 (mmHg), *p*_5_ = 0 (mmHg) etc.). This setup was particularly useful to analyze the impact of changes in membrane morphology on channel gating.

### Analysis of Experimental Data

#### Basic Analysis of Channel Kinetics

Basic analysis of single-channel currents assumed the evaluation of open-state probability (*p*_op_) and mean dwell time of open (conducting) and closed (nonconducting) states (*τ*_op_ and *τ*_cl_, respectively), as given in (Mercik et al. 1999). Besides the most simplified approach where only two macrostates are considered (open-closed), we have also checked whether the mechanical tension unravels some additional substates in the multistate Markovian model of gating kinetics (Geng and Magleby, 2015). Typically, 3–4 open states and 5–6 closed states can model BK channel gating (Geng and Magleby, 2015), but in the low-calcium regime (as in our experiments) one should rather expect 2–3 open and 3 closed states (Blatz and Magleby, 1986; Magleby and Palotta 1983) as presented in the scheme below.

1 $$\begin{array}{*{20}{c}} {{C_5}} \\ ~ \\ ~ \end{array}~~~~\begin{array}{*{20}{c}} {\begin{array}{*{20}{c}} \leftrightarrow \\ ~ \\ ~ \end{array}}&{\begin{array}{*{20}{c}} {{C_4}} \\ \updownarrow \\ {{O_2}} \end{array}~~~~~\begin{array}{*{20}{c}} \leftrightarrow \\ ~ \\ \leftrightarrow \end{array}}&{\begin{array}{*{20}{c}} {{C_3}} \\ \updownarrow \\ {{O_1}} \end{array}} \end{array}$$

Based on dwell-time distributions, we have estimated a minimal number of states in Markovian model that can describe the kinetics of the BK channel gating at a given external condition (membrane potential and suction). Each substate (*C*_*i*_ or *O*_*i*_) should contribute to the appropriate dwell-time distribution (*f*(*t*)) as an exponential component according to formula (Geng and Magleby 2015; McManus and Magleby 1988, 1989):

2 $$f\left( t \right)=~\mathop \sum \limits_{{i=1}}^{N} \frac{{{a_i}}}{{{\tau _i}}} \times \exp \left( { - \frac{t}{{{\tau _i}}}} \right),$$

where *N* is a number of substates within a manifold of a given macrostate (open or closed), *a*_*i*_ describes the fraction of the total area of the dwell-time distribution contributed by the *i*-th exponential, *τ*_*i*_ is a time constant corresponding to the *i*-th exponential.

The experimental open and closed dwell-time distributions were fitted by a sum of exponentials, estimating the number of substates describing channel kinetics according to the Markovian model. If some changes in the number of substates, areas or time scales from Eq. (2) occur at different levels of membrane suction, it can allow for some inferences about the impact of this stimulus on channel gating—accessibe channel states and probability of their occurence.

#### Nonlinear Analysis of the Series of Dwell Times of Subsequent Channel States

For comprehensive characterization of complex dynamics hidden behind the process, the set of nonlinear measures calculated were Hurst Exponent, Sample Entropy (SE), and Correlation Dimension.

As a first quantifier of internal properties of the signal obtained from single-channel recordings, Hurst exponent was evaluated as a measure of long-range correlations in the series of the dwell times of subsequent channel states. There are several algorithms for calculating this feature (Kirichenko et al. 2011). In this work, we compare the best-known Rescaled Range Analysis or R/S method (Gilmore et al. 2002; Kantelhardt et al. 2001; Kirichenko et al. 2011; Semmlow and Griffel 2014) with the results obtained by the Detrended Fluctuation Analysis (DFA) (Ihlen 2012). Both algorithms determine a level of self-similarity, but the latter takes into account the nonstationarity of a data. The brief description of DFA procedure is presented below.

The procedure starts with calculation of profile *y*_*i*_ as the cumulative sum of the data *x*_*i*_ with the subtracted mean $\overline {x} :{y_i}=\left[ {{x_k} - \overline {x} } \right]$. Next, signal *y*_*i*_ is split into *N*_*s*_ nonoverlapping segments of size *s*. For all the segments of number *v*, the local trend $y_{{v,i}}^{m}$ is calculated by means of the least-square fit of order *m*. In the penultimate step, the variance *F*^*2*^ is determined as a function of the segment length *s* for each segment *v* separately (3).

3 $${F^2}\left( {s,v} \right)=~\frac{1}{s}\mathop \sum \limits_{{i=1}}^{s} {\left( {y_{{v,i}}^{m} - {y_{v,i}}} \right)^2}$$

Finally, the Hurst exponent is estimated as the slope of the regression line of the double-logarithmic dependence log(*F*) ∝ *H*log(*s*). The values of *H* exponent are interpreted as follows: *H* ϵ (0;0.5) indicates antipersistency of the signal, *H* = 0.5 is assigned to the uncorrelated noise, and *H* ϵ (0.5;1) characterizes the time series with long-term positive autocorrelation i.e., persistent data (Kantelhardt et al. 2001, 2002).

##### Sample Entropy (SE)

The idea of entropy in the context of biological data analysis was derived from information theory that was first presented by Claude Shannon in 1948 (Shannon 1948). The Shannon entropy characterizes the amount of information in a signal. Further development in this field led to implementation of many different notions of entropy, including the measures that refer to the rate at which signal loses or gains information (Semmlow and Griffel 2014). These measures, like Approximate Entropy (*AE*) or its updated version Sample Entropy, give an insight about the level of uncertainty in the context of signal dynamics. In other words, they are measures of complexity of the series. The algorithm of *SE* utilizes the fact that we can predict the level of complexity by calculating how a representative subseries is repetitive throughout the whole signal. *SE* is defined by the negative natural logarithm of the conditional probability that two similar *m*-points long vectors are still similar if we extend these vectors to *m* + 1 points, at the given tolerance threshold *r*. The *r* value is arbitrarily chosen from the range of 10–20% of the standard deviation of the series. For a step-by-step investigation of the *SE* algorithm, see Costa et al. (2005). In this paper, we set together two kinds of results, that differ from each other by Euler or Chebyshev definitions for the distance functions in the *SE* algorithm.

##### Multiscale Entropy

*MSE* together with the *DFA* method characterizes the properties of the system for the variety of time scales. *MSE* is a direct extension of SE over multiple time scales. The set of series with different scales is obtained by coarse-graining procedure, which is the simple average of data points for each nonoverlapping segments of points. The consecutive time series of different scale factors $y_{j}^{\tau }$ are represented by Eq. (4) where *N* is assigned to the length of the series and *τ* stands for the actual scale factor (Costa et al. 2005).

4 $$y_{j}^{\tau }=~\frac{1}{\tau } \times \mathop \sum \limits_{{i=\left( {i - 1} \right)\tau +1}}^{{j\tau }} {x_i}\quad \quad 1 \leqslant j \leqslant \frac{N}{\tau }~.$$

##### Correlation Dimension

The correlation dimension *D*_corr_ is another measure strongly related to the complexity of the system. It is the geometric average of the generalized fractal dimension (Theiler 1990). This feature characterizes the geometry of chaotic attractors and is defined using the correlation sum *C*(*r*), the mean probability that the states at two different times are close on the chosen threshold distance *r*. The equation that relates the mean probability with correlation dimension is presented below.

5 $$C\left( r \right)~ \propto ~{r^{{D_{corr}}}}.$$

For more details and description of the Grassberger–Procaccia algorithm used in the calculation of *D*_corr_, see (Grassberger and Procaccia 1983; Kember and Fowler 1992).

#### Statistics

In the experimental mode of gradual monotonic change of membrane suction (studies of mechanosensitivity), 10 to 15 independent single-channel patch-clamp measurements were recorded for each voltage and membrane suction level. In alternate suction mode (investigations of membrane fatigue effects on channel activity), the series of experiments in alternate mode were performed 6 times independently at both voltages. In both cases, every single patch-clamp recording comprised *N* = 6 × 10^5^ current values.

The open-state probabilities (*p*_op_) and mean dwell times of open (conducting) and closed (nonconducting) states (*τ*_op_ and *τ*_cl_, respectively) are given as the average values from all recordings at given external conditions.

For distinct membrane suctions at *U*_m_ = − 50 (mV) and *U*_m_ = + 50 (mV), the average values of the nonlinear analysis parameters are calculated for the three representative individual measurements, each consisting of 5000 data points. The length of those datasets was dictated by the maximum number of data points that could be acquired experimentally without the significant impact of the typical membrane artifacts (like the last channel’s state recorded in some experiments—outstandingly long closure which, probably, indicates channel’s inactivation). In turn, those artifacts would have the dominant impact on the statistical description, especially in the case of fluctuation analysis. Error bars visible in the figures correspond to the standard deviation of the mean (SEM) for the kinetic and nonlinear analysis parameters. This work presents the general properties which can characterize gBK channel mechanosensitivity and its dependence on the membrane morphology.

## Results

### Mechanosensitivity of BK Channels

**Fig. 1:**
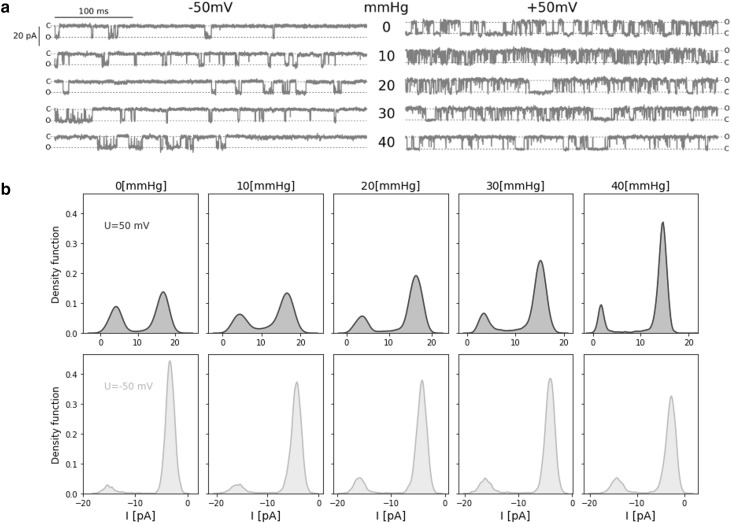
**a** The samples of the original signal of ionic current recorded from a single gBK channel over the range of membrane suctions at fixed electric potential (*U*_m_ = − 50 (mV) on the left side and *U*_m_ = + 50 (mV) on the right side). Dashed lines indicate open (O) and closed (C) states of the channel. **b** Probability density function of single-channel current (*I*) at given membrane suction and potential

**Fig. 2:**
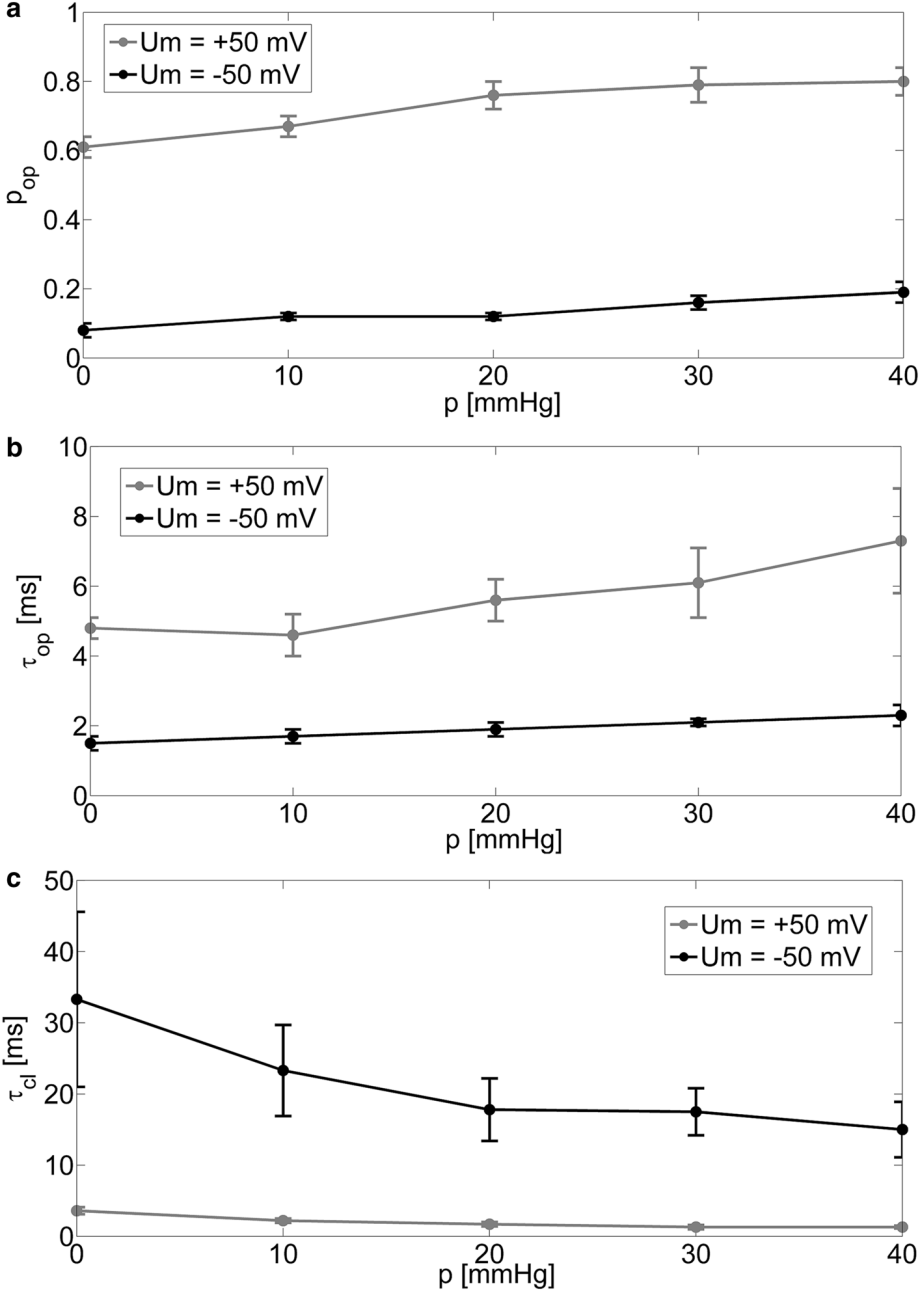
Experimental dependencies of open-state probability (*p*_op_) and mean dwell time of open and closed states (*τ*_op_, *τ*_cl_) on the difference in pressure on either side of membrane (*p*) (parts **a, b, c**, respectively) obtained at membrane depolarization and hyperpolarization

The obtained results show considerable mechanosensitivity (Figs. 1, 2) for both membrane hyperpolarization and depolarization. Open-state probability increases with membrane suction at positive potential; however, the increase is higher (for full range of the suction it is Δ*p*_op_ = 0.11 at *U*_*m*_ = − 50 (mV) and Δ*p*_op_ = 0.19 at *U*_m_ = + 50 (mV)). Analogous tendency is observed within mean dwell times of open state, namely, the appropriate increments are Δ*τ*_op_ = 0.8 at *U*_m_ = − 50 (mV) and Δ*τ*_op_ = 2.5 (ms) at *U*_m_ = + 50 (mV). Mean dwell times of closed states decrease with raising difference in pressure on the opposite sides of membrane. The decrease is evident at membrane hyperpolarization Δ*τ*_cl_ = − 18.3 (ms) and significantly lower at membrane depolarization Δ*τ*_cl_ = 2.5 (ms).

Although mechanical strain activates BK channel (Figs. 1, 2), the dwell-time distributions can be described by a sum of two exponential in the case of channel openings and three exponentials for closings at all analyzed levels of membrane suction. Thus, regardless of membrane tension, 5-state Markovian model (1) is applicable to describe channel kinetics.

**Fig. 3:**
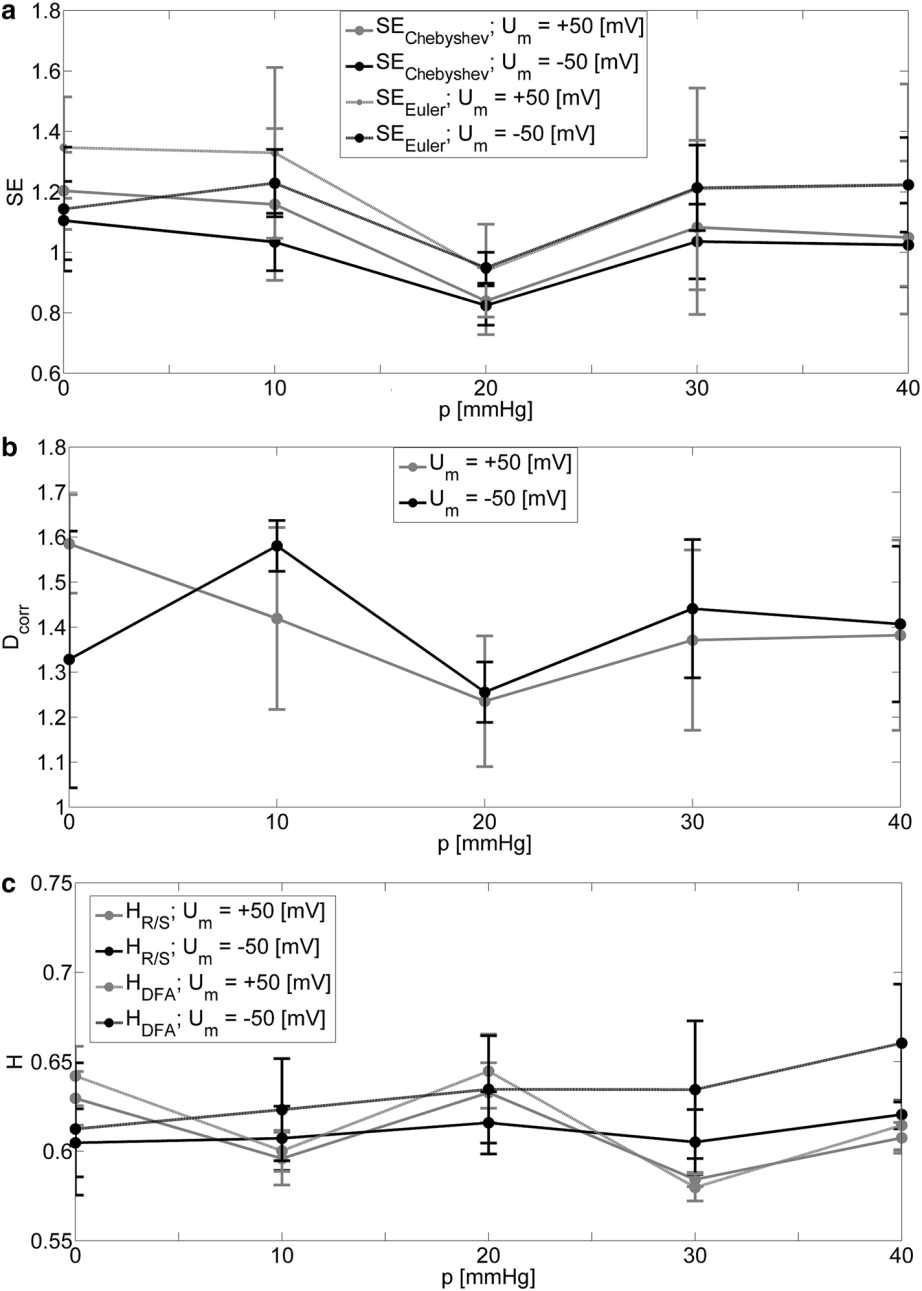
Values of parameters of nonlinear analysis as a function of membrane suction at membrane hyperpolarization and depolarization

The sets of parameters of nonlinear analysis calculated in the analyzed range of membrane suction at *U*_m_ = − 50 (mV) and *U*_m_ = + 50 (mV) are presented in Table 1 and Fig. 3. Table 1 includes sample entropy parameters (SE_Chebyshev_, SE_Euler_), correlation dimension (*D*_corr_), and Hurst exponent calculated by the standard R/S (*H*_R/S_) and DFA (*H*_DFA_) method.

**Table 1:** The average values of parameters of nonlinear analysis calculated for *U*_m_ = − 50 (mV) and *U*_m_ = + 50 (mV)

*p* (mmHg)	*U*_m_ = − 50 (mV)					*U*_m_ = + 50 (mV)				
	SE_Chebyshev_	SE_Euler_	*D*_corr_	*H*_R/S_	*H*_DFA_	SE_Chebyshev_	SE_Euler_	*D*_corr_	*H*_R/S_	*H*_DFA_
0	1.11	1.14	1.33	0.61	0.61	1.20	1.35	1.59	0.63	0.64
10	1.03	1.23	1.58	0.61	0.62	1.16	1.33	1.42	0.60	0.60
20	**0.82**	**0.95**	**1.26**	0.62	0.64	**0.84**	**0.94**	**1.24**	0.63	0.65
30	1.04	1.21	1.44	0.61	0.63	1.08	1.21	1.37	0.58	0.58
40	1.02	1.22	1.41	0.62	0.66	1.05	1.22	1.38	0.61	0.61

Bold values indicate minimal values of the measures of complexity

**Fig. 4:**
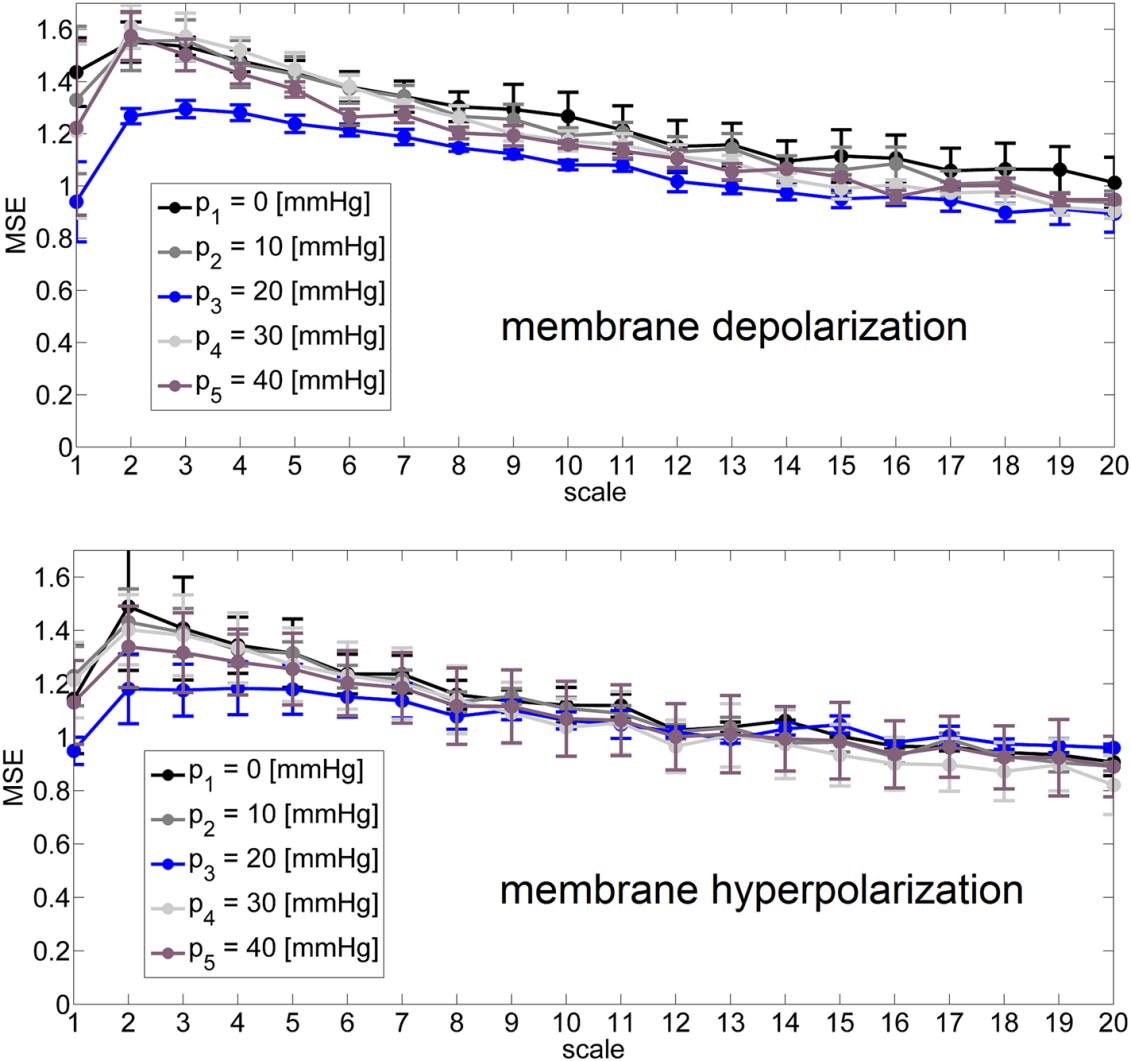
Results of multiscale entropy analysis at membrane hyperpolarization and depolarization

The values of Hurst parameter, calculated by any of the chosen techniques are close to 0.6 for the all analyzed cases. This indicates the presence of long-range correlations in the analyzed signals. However, there is a lack of any significant tendency for the change of the Hurst exponent with membrane strain. For the measures of complexity, we can observe significant decrease of SE and *D*_corr_ values for *p* = 20 (mmHg) in the case of both, positive and negative voltages (as presented by bold values in the Table 1). This tendency is also clearly visible in Fig. 4 where the *SE* parameters over the range of scales are presented. The curves assigned to the *p* = 20 (mmHg) significantly stand out from the others at the level of small range of scales *s* ϵ {1,2,3,4,5}. This fact also provides the additional insight that the information in the signals under consideration is contained in rather short time scales.

Considering the states of membrane polarization individually, for positive voltage [*U*_m_ = + 50 (mV)], the highest value of SE and *D*_corr_ occurs for the lowest pressure, and decreases, respectively, with the increasing difference in pressure on the opposite sides of membrane to the *p* = 20 (mmHg). Quite different results are obtained for the negative voltage [*U*_m_ = − 50 (mV)], where the slight increase of complexity measure can be observed for *p* = 10 (mmHg).

### Effects of Membrane “Fatigue” on BK Channels’ Activity

**Fig. 5:**
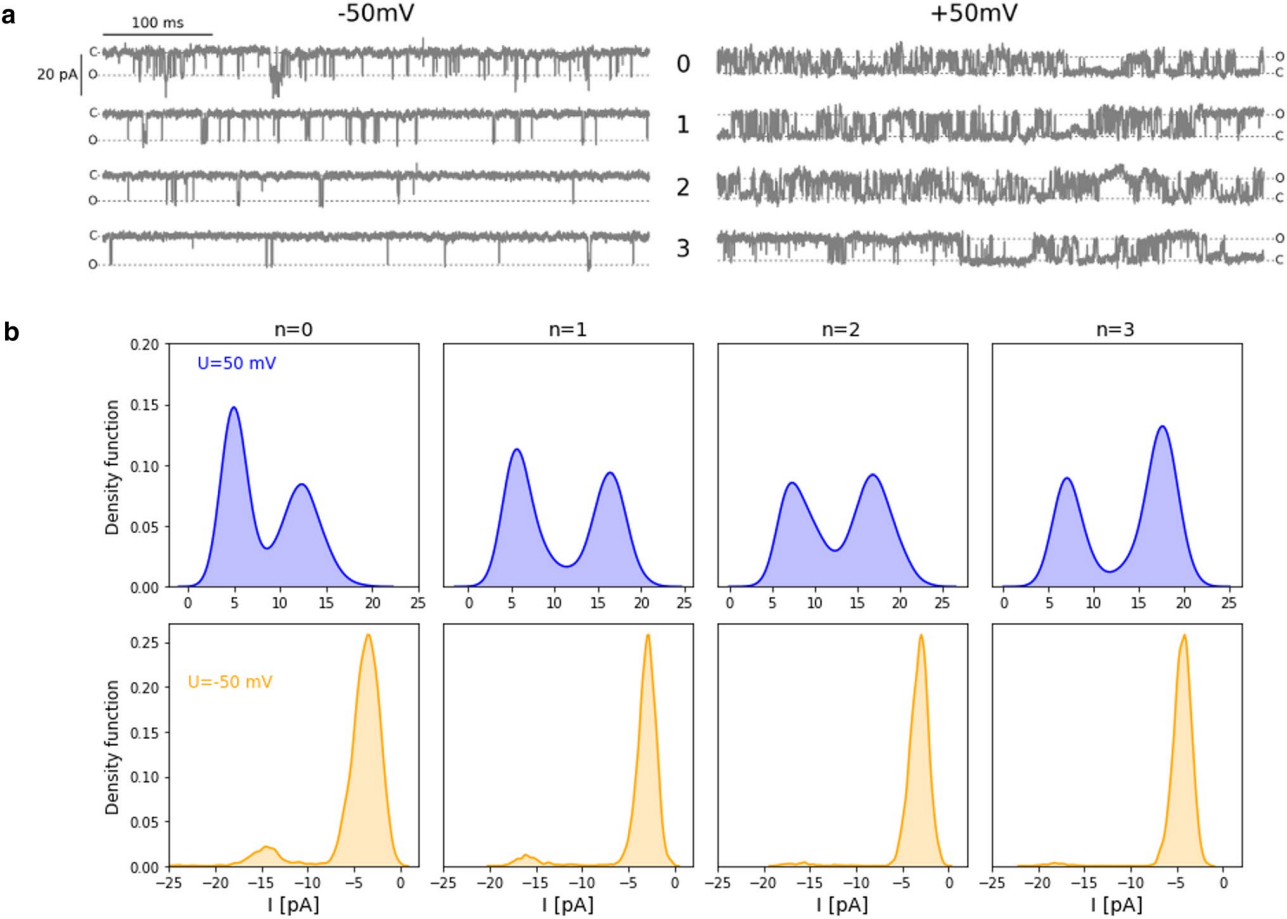
**a** The samples of the original signal of ionic current recorded from a single gBK channel after a given number of prolonged membrane suctions (*n* ϵ {0,1,2,3}) at fixed electric potential (*U*_m_ = − 50 (mV) on the left side and *U*_m_ = + 50 (mV) on the right side). Dashed lines indicate open (O) and closed (C) states of the channel. **b** Probability density function of single-channel current (*I*) after *n* prolonged membrane suctions and at given membrane potentials

**Fig. 6:**
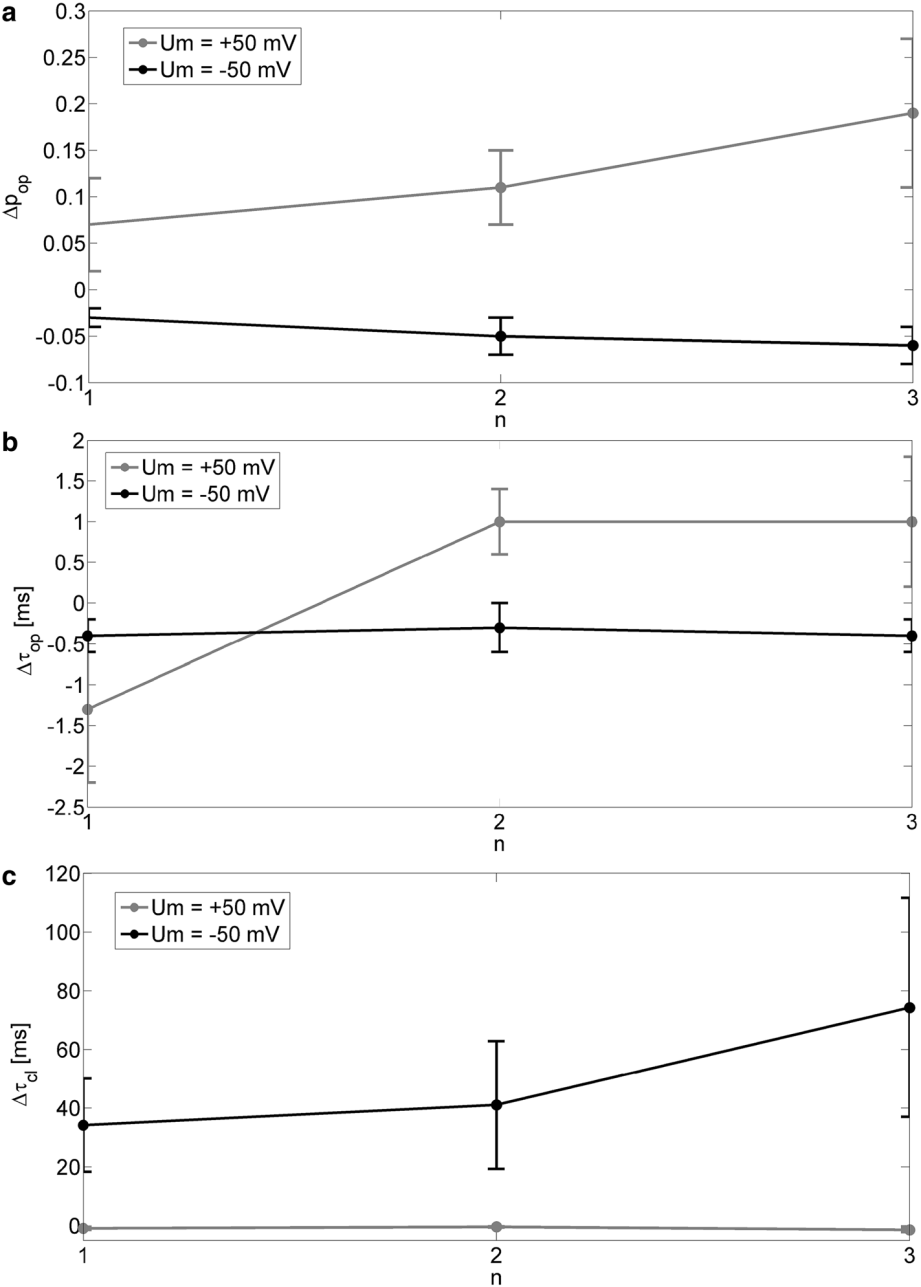
Effects of membrane stimulation by series of *n* prolonged pressure impulses on channel activity (measured by open-state probability and mean dwell times of open and closed states of a channel) at membrane hyperpolarization and depolarization

In measurements carried out in an alternate suction mode, channel activity turned out to be dependent on the number of measurements under membrane suction (*n*) separating the recordings where membrane was in a resting state [*p* = 0 (mmHg)] Figs. 5 and 6. The impulses of prolonged membrane suction exerted other effects in the negative and positive voltage regime. When the channel was voltage-activated, repeated suction pulses caused increase in open-state probability of 19%. In turn, at negative membrane potential *p*_op_ decreased with *n* of about 6%. The changes in mean dwell times of open and closed states (Fig. 6b, c) also show that the membrane “fatigue” induced by suction of a membrane patch causes apparent channel activation at high voltage and an opposite effect at low voltage.

We also checked whether the mentioned effects are time-dependent. We examined whether during each recording (at both voltages and after each number of suction impulses) the values of *p*_op_, *τ*_op_, and *τ*_cl_ remained constant. No relaxing effect was noticed within all series.

**Fig. 7:**
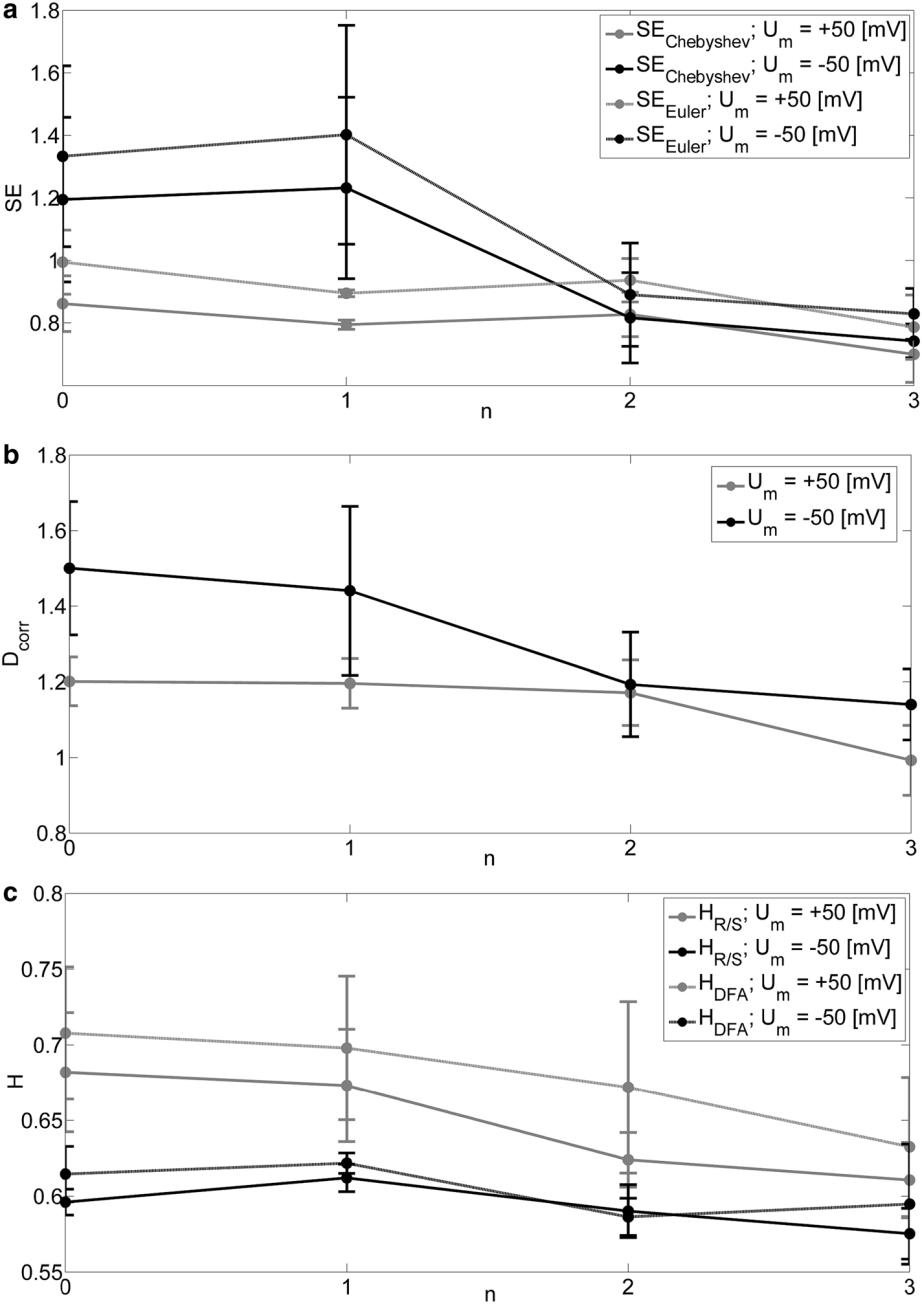
Values of parameters obtained by nonlinear analysis as a function of a number of suction impulses (*n*) at membrane hyperpolarization and depolarization

The values of parameters obtained by nonlinear analysis for the different levels of membrane fatigue are presented in Table 2 and Fig. 7. It is necessary to emphasize that there are around 600 data points used for this calculation at *U*_m_ = − 50 (mV). This particular choice was dictated by the common permanent inactivation of a channel, which reduced a number of recognized states after several impulses. We can observe less distinct differences between the successive measurements than for the case with effect of the pressure. The cause of this difference may lie in the short length of series. However, the decrease of the average values of complexity measures is clearly visible between the most distant measurements (0 and 3 impulses) for both depolarization and hyperpolarization.

**Table 2:** The average values of parameters obtained by nonlinear analysis after *n* impulses of membrane suction calculated for *U*_m_ = − 50 (mV) and *U*_m_ = + 50 (mV)

*n*	*U*_m_ = − 50 (mV)					*U*_m_ = + 50 (mV)				
	SE_Chebyshev_	SE_Euler_	*D*_corr_	*H*_R/S_	*H*_DFA_	SE_Chebyshev_	SE_Euler_	*D*_corr_	*H*_R/S_	*H*_DFA_
0	1.19	1.33	1.50	0.60	0.61	0.99	1.16	1.20	0.68	0.71
1	1.23	1.40	1.44	0.61	0.62	0.79	0.90	1.20	0.67	0.70
2	0.82	0.89	1.19	0.59	0.59	0.83	0.94	1.17	0.62	0.67
3	0.74	0.83	1.14	0.58	0.59	0.70	0.79	0.99	0.61	0.63

## Discussion

Our analysis broadened the physiological characterization of gBK channels. The obtained results indicated that the gBK channels’ activity is affected by mechanical strain of cell membrane both at membrane depolarization and hyperpolarization, but some differences in kinetic characteristics are visible on quantitative level for voltage-activated and nonactivated channels.

Although mechanical strain of membrane does not reveal any additional substates within open and closed manifolds according to the Markov model, it does not mean that the number of available channel’s conformations is retained from spatial and energetic point of view. When membrane suction is applied, one can observe that the calculated measures of dynamical complexity, sample entropy parameters (SE_Chebyshev_, SE_Euler_) and correlation dimension (*D*_corr_), exhibit nonmonotonic dependence on pressure difference on the opposite sides of membrane patch with a minimum at *p* = 20 (mmHg) both at positive and negative membrane potential. The general tendency of the considered parameters is that they reach higher values for resting patches than in the case of highly strained ones. Thus, the values of SE_Chebyshev_, SE_Euler_ and *D*_corr_ indicate relatively low complexity of the dwell time series at strong mechanical tension within membrane (at strong suction). In terms of gating dynamics this result can suggest that smaller number of channel’s conformations is spatially or energetically available when cell membrane is mechanically stressed than in case when no suction is applied on the membrane. Minima of the mentioned parameters at *p* = 20 (mmHg) are not surprising. Due to the high complexity of channel’s structure (Cox 2007; Cui et al. 2008) and the possible interactions with its membrane surroundings (Lee 2004; Sachs 2010; Suchyna et al. 2009) the response of the characteristics of channel dynamics to membrane strain may be nonlinear.

As one can see, in the current research, we showed the usability of analysis by nonlinear methods for characterization of ion-channel recordings. This kind of analysis, which is uncommon in the field, has a clear interpretation and allows for some inferences in a nanoscale—referring to conformational dynamics of channel proteins.

The long-range memory is conserved in the full range of pressure differences applied on membrane in our experiments and stimulation of cell membrane by pressure impulses (regardless of the algorithm used to calculate the Hurst exponent) (Figs. 3c, 7c). Thus, mechanical strain is another stimulus, like voltage, Ca^2+^ concentration, temperature (Barbosa et al. 2007; Wawrzkiewicz et al. 2012; Wawrzkiewicz-Jałowiecka et al. 2017), that does not affect the presence of long-range correlations in series of dwell times of subsequent channel’s states.

In the current research, we also investigated how influencing membrane morphology by relatively long impulses of membrane suction leads to the changes in channel activity. We described that effect quantitatively. Slight activation of the channel was observed at membrane depolarization (after three prolonged suctions of the membrane *p*_op_ increased of 19%) and its deactivation at membrane hyperpolarization (after three prolonged suctions of the membrane *p*_op_ decreased of 6%). Thus, the impact of possible loosening membrane structure on the channel depends on the position of voltage sensor within the membrane. Loosening of membrane structure seems to affect the conformational dynamics of the channel by increasing probability of retaining in a preferred conformation at a given membrane potential, which is open at high voltages and closed at negative ones. This inference is confirmed by the results of signal complexity analysis. Namely, one can observe decrease of sample entropy and correlation dimension with loosening of membrane structure (Fig. 7a,b).

Both mechanosensitivity of gBK channels and their dependence on the membrane morphology (whether or not cytoskeleton is anchored to membrane) described in the current work may contribute to their physiological role by supporting extensive migrating behavior of glioblastoma cells (Weaver et al. 2006). Especially, at membrane depolarization, one can observe considerable increase in channel activity, improving the effectiveness of K^+^ transport through the cell membrane evoked by mentioned stimuli. Mechanosensitivity of gBK, stated in this work, can affect mechanostransduction during the rapid shape and volume changes of glia cells during their extensive migration.
